# The Influence of Chicken Egg Lysozyme or Zinc-Bacitracin Antibiotic on the Growth Performance, Antibacterial Capacity, Blood Profiles, and Antioxidative Status of Rabbits: A Comparative Study

**DOI:** 10.3390/ani11061731

**Published:** 2021-06-10

**Authors:** Mahmoud H. EL-Deep, Khairy A. Amber, Yahia Z. Eid, Salama Mostafa Aboelenin, Mohamed Mohamed Soliman, Mohamed S. Sakr, Mahmoud A. O. Dawood

**Affiliations:** 1 Animal Production Research Institute, Sakha Station, Kafr El-Sheikh 33717, Egypt; mheldeep1980@yahoo.com (M.H.E.-D.); msqr977@gmail.com (M.S.S.); 2 Department of Poultry Production, Faculty of Agriculture, Kafrelsheikh University, Kafr El-Sheikh 33516, Egypt; khairy.amber@agr.kfs.edu.eg (K.A.A.); yahyaze@gmail.com (Y.Z.E.); 3 Biology Department, Turabah University College, Taif University, P.O. Box 11099, Taif 21944, Saudi Arabia; s.aboelenin@tu.edu.sa; 4 Clinical Laboratory Sciences Department, Turabah University College, Taif University, P.O. Box 11099, Taif 21944, Saudi Arabia; mmsoliman@tu.edu.sa; 5 Department of Animal Production, Faculty of Agriculture, Kafrelsheikh University, Kafr El-Sheikh 33516, Egypt

**Keywords:** feed additives, antibiotics, rabbits, growth performance, gene expression

## Abstract

**Simple Summary:**

Despite the beneficial role of antibiotics in reducing bacterial infection in rabbits, there is an indirect harmful influence on human health. Thus, replacing antibiotics with friendly alternatives is a suitable strategy to protect the performance and welfare of rabbits. This study aimed at comparing the effects of including dietary egg lysozyme and zinc bacitracin antibiotic (ZnB) on the productivity and health conditions of rabbits. The results show a marked enhancement of the growth performance, antibacterial capacity, blood health, and antioxidative status in rabbits treated with egg lysozyme compared with those treated with ZnB. Thus, using egg lysozyme is recommended to replace the usage of ZnB in rabbit production.

**Abstract:**

Dietary egg lysozyme has beneficial roles in the growth performance and health conditions of animals. The study was performed using 90 multicolored rabbits in three groups (each replicate with thirty rabbits). In the control group, rabbits were fed a diet without zinc bacitracin (ZnB) or egg lysozyme, while the second and third groups were treated with ZnB and lysozyme additive at 100 mg/kg, respectively. After eight weeks, the final weight and body weight gain (BWG) of rabbits fed dietary egg lysozyme and ZnB additives were meaningfully increased (*p* < 0.05). Nevertheless, the feed conversion ratio (FCR) was markedly decreased by dietary egg lysozyme and ZnB (*p* < 0.05). Interestingly, dietary egg lysozyme resulted in higher final weight and BWG and lower FCR than rabbits treated with ZnB (*p* < 0.05). Rabbits treated with egg lysozyme and ZnB additives had markedly lower populations of *Clostridium* spp. and *Escherichia coli* (*p* < 0.05) compared with the control. However, the counts of *Lactobacillus* and total bacteria were meaningfully increased in the the intestines of rabbits treated with egg lysozyme and ZnB (*p* < 0.05). The blood total protein and globulin of rabbits fed dietary egg lysozyme and ZnB additives were meaningfully increased (*p* < 0.05). Blood creatinine was significantly lowered by dietary egg lysozyme compared with the control and ZnB-treated rabbits (*p* < 0.05). The levels of blood urea, ALT, and AST were markedly lowered (*p* < 0.05) by dietary egg lysozyme and ZnB. The gene expressions of superoxide dismutase 1 (*SOD1*) and glutathione peroxidase (*GPX*) in the liver of rabbits fed dietary egg lysozyme and ZnB additives were markedly upregulated (*p* < 0.05) compared with the control. Dietary egg lysozyme resulted in higher expression of *SOD1* and *GPX* genes than rabbits treated with ZnB (*p* < 0.05). In conclusion, the inclusion of egg lysozyme could replace the inclusion of ZnB in the diets of rabbits.

## 1. Introduction

Rabbit meat is widely consumed as a tasty and nutritious protein source with high commercial value [1]. However, rabbits are sensitive to infection with pathogenic bacteria, primarily when raised in stressful conditions [2]. In this sense, antibiotics have been widely used as protective chemotherapy and immunostimulant agents [3,4]. Several antibiotics were applied in livestock breeding, such as zinc-bacitracin antibiotic (ZnB), which has marked growth-promoting efficacy [5]. ZnB is a prophylaxis antibiotic growth promotor (AGP) with a high potential to regulate the intestinal microbiota and wall structure associated with a high content of polypeptides [6]. Despite the beneficial role of ZnB antibiotics against infection, the continuous inclusion of ZnB induces the suppression of natural immunity, the disruption of natural intestinal microbiota, and high accumulation of antibiotic derivatives in animal flesh [4]. In addition, high addiction to ZnB results in increased resistance of the microbial populations in the intestines of animals, making ZnB harmful and useless [7]. Therefore, the application of AGP is banned by the European union and several countries around the globe and has been replaced with natural friendly functional substances (e.g., probiotics, prebiotics, and phytobiotics) [8,9,10,11]. 

Alternatively, lysozyme extracted from eggs was approved as an effective natural growth-promoting and antibacterial agent in rabbit and broiler production [3,12]. Lysozyme can deactivate N-acetylmuraamic acid and N-acetylglucosamine residues in Gram-positive bacteria [13]. In addition, lysozyme is has anti-inflammatory and immunostimulant properties, making it a great candidate to replace antibiotics in the animal production industry [14]. The treatment with egg lysozyme increased the abundance of beneficial microorganisms and lowered the harmful populations in the gut of animals [15,16,17]. The inclusion of dietary lysozyme resulted in the enhanced diversity of intestinal microbiota, growth performance, immune, and antioxidative responses of broiler chickens [12]. In addition, weaned pigs treated with dietary lysozyme had improved growth performance, diversity of beneficial intestinal microbiota, health of intestinal barriers, and immune response [15,16,17,18,19]. Interestingly, the inclusion of lysozyme resulted in improved growth performance, intestinal health, humoral and cellular immunity, antioxidative response, and disease resistance in finfish species [20,21,22,23,24]. In rabbits, dietary lysozyme resulted in enhanced growth rate, blood health, and antibacterial capacity [3]. As dietary egg lysozyme has already been tested as a growth promotor in rabbits, the current study aimed at comparing the beneficial roles of dietary egg lysozyme with ZnB to propose the possible strategies for reducing AGP in the rabbit industry.

Both ZnB and lysozyme have antibacterial capacity against Gram-positive bacteria and can actively enhance the growth performance and well-being [14,25]. However, ZnB is an antibiotic-sourced additive and can be replaced with natural active substances such as egg lysozyme. It can be hypothesized that using a natural antibacterial agent (egg lysozyme) can replace AGP (ZnB) usage in the rabbit industry. Thus, this study investigated the comparison between dietary ZnB and egg lysozyme as functional additives in the diets of growing rabbits. The study detected the effect of ZnB or egg lysozyme on the growth performance, antibacterial capacity, blood biochemical traits, and antioxidative response in rabbits.

## 2. Materials and Methods

### 2.1. Experimental Design 

The study was performed by using APRI-Line weaning male rabbits aged five weeks and weighed 611.32 ± standard deviation (0.36) g/animal. In the farms of Sakha Poultry Research Station and Laboratories, Kafrelsheikh, Animal Production Research Institute, Egypt, 90 multicolored rabbits were distributed into three groups (each group with thirty rabbits). Rabbits were individually housed in galvanized batteries (60 cm × 40 cm × 35 cm) provided with automatic nipples and feeders. Rabbits were kept under controlled hygienic conditions (14 h of light/day) and fed the prepared, tested rations for eight weeks. Rabbits were fed ad libitum, and freshwater was available in each cage through automatic nipples. The temperature and relative humidity were 18–20 °C and 55–65% during the trial, respectively. Rabbits in the control group were fed a diet without zinc bacitracin (ZnB) or egg lysozyme, while the second and third groups were treated with ZnB and lysozyme additive at 100 mg/kg, respectively. The doses of egg lysozyme and ZnB were proposed by following El-Deep et al. [3] and Thema et al. [5], respectively. The composition of the basal diet is presented in Table 1, which was formulated by following National Research Council (NRC) [26], and the composition was checked by following the standard method of Association of Official Analytical Chemists (AOAC) [27]. The lysozyme was prepared by following Ibrahim et al. [28] with a purity of 10%, while ZnB was provided by General Pharma Company^TM^ (10% purity, Kafrelsheikh, Egypt). The ingredients were pelleted and then mixed with egg lysozyme and ZnB in the presence of molasses to avoid the loss of additives’ functionality and concentration. The feed intake and live body weight were recorded on two fixed days weekly, and then body weight gain and feed conversion ratio were calculated.

### 2.2. Microbial Enumerations

Samples from the cecum were chosen individually from six rabbits from each group, and cecum contents were obtained after slaughtering and filtrated to estimate cecum microflora. Total bacterial count, *Escherichia coli*, *Clostridium*, and *Lactobacillus* count were performed according to Collins and Lyne [29].

### 2.3. Blood Sampling and Biochemistry Examination 

The blood was collected from the lateral ear vein of slaughtered rabbits (9 rabbits per group). The blood was left to clot at room temperature and then centrifuged at 3000 rpm for 15 min. Sera were then separated and stored at −20 °C in aliquots for individual biochemical estimations. 

The serum was used to detect alanine aminotransferase (ALT), aspartate aminotransferase (AST), total proteins, albumin, creatinine, urea, triglycerides, and cholesterol were detected according to the manufacturer’s protocol using commercial kits (Diamond Diagnostic, Dokki, Giza, Egypt). Globulins concentration in serum was computed by subtracting albumin concentration from total proteins, while the albumin to globulin ratio (A/G) was calculated according to Kaneko et al. [30]. 

### 2.4. Gene Expression

Part of the livers from 6 rabbits per group were collected and immediately frozen in liquid nitrogen. Total RNA was extracted by easy-RED Total RNA Extraction Kits (iNtRON Biotechnology, Inc., Gyeonggi-do, Korea) using the manufacturer procedure. The integrity of RNA was checked by agarose gel electrophoresis, while a NanoDrop spectrophotometer detected the purities and quantities of the samples. The first-strand cDNA was obtained using a kit for HiSenScript cDNA (iNtRON Biotechnology, Inc., Gyeonggi-do, Korea). Specific primers were used to amplify chosen genes with glyceraldehyde 3-phosphate dehydrogenase (GAPDH) as a housekeeping gene that was stable among the sample groups (Table 2). The mRNA expression was performed using a Stratagene MX3005P real-time PCR (Agilent Technologies, CA, USA) and TOPrealTM PreMIX SYBR Green qPCR master blend (Enzynomics, Daejeon, Korea) following the suggestions of the manufacturer. MxPro QPCR Software was used. The relative concentrations of gene expression were assessed using the 2^−ΔΔct^ technique as outlined in Pfaffl [31].

### 2.5. Statistical Analysis

The obtained data were subjected to one-way analysis of variance using SPSS (version 22, Armonk, NY, USA). Differences within means of treatments were tested by Tukey’s test at *p* < 0.05. 

## 3. Results

### 3.1. Growth Performance

The final weight and body weight gain (BWG) of rabbits fed dietary egg lysozyme and ZnB additives were meaningfully increased (*p* < 0.05) compared with the control (Table 3). Nevertheless, the feed conversion ratio (FCR) and feed intake (FI) were markedly decreased by dietary egg lysozyme and ZnB (*p* < 0.05). Interestingly, dietary egg lysozyme resulted in higher final weight and BWG and lower FCR and FI than rabbits treated with ZnB (*p* < 0.05) (Table 3).

### 3.2. Cecum Microbial Load

Rabbits treated with egg lysozyme and ZnB additives had markedly low populations of *Clostridium* spp. and *Escherichia coli* (*p* < 0.05) compared with the control (Table 4). However, the counts of *Lactobacillus* and total bacterial were meaningfully increased in the cecum of rabbits treated with egg lysozyme and ZnB (*p* < 0.05). The inclusion of egg lysozyme resulted in higher counts of *Lactobacillus* and total bacterial count than dietary ZnB (*p* < 0.05) (Table 4). 

### 3.3. Blood Biochemistry

The blood total protein and globulin of rabbits fed dietary egg lysozyme and ZnB additives were meaningfully increased (*p* < 0.05) compared with the control (Table 5). Dietary egg lysozyme resulted in higher blood total protein and globulin than rabbits treated with ZnB (*p* < 0.05). Blood creatinine was significantly lowered by dietary egg lysozyme compared with the control and ZnB-treated rabbits (*p* < 0.05) (Table 5). The levels of blood urea, ALT, and AST were markedly lowered (*p* < 0.05) by dietary egg lysozyme and ZnB (Table 5). Rabbits fed lysozyme had a lower AST level than rabbits fed ZnB (*p* < 0.05). No meaningful impacts of egg lysozyme or ZnB were seen on the blood cholesterol and triglycerides (*p* > 0.05) (Table 5).

### 3.4. Antioxidant Related Gene Expression

**Figure 1:**
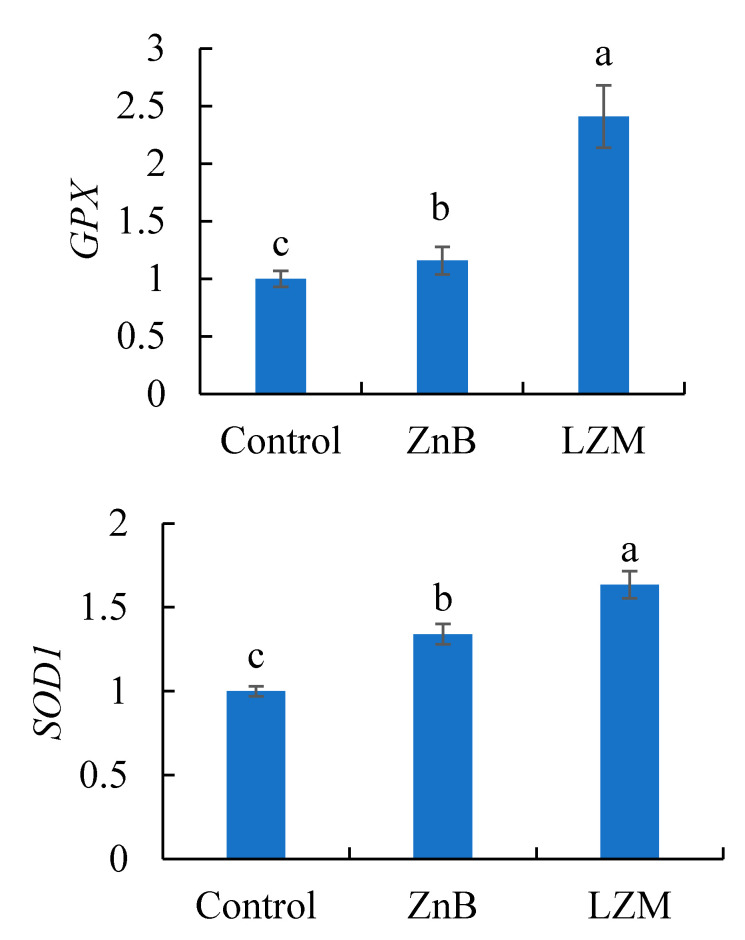
Effect of chicken egg lysozyme (LZM) and zinc bacitracin antibiotic (ZnB) on the relative gene expression of superoxide dismutase (*SOD1*) and glutathione peroxidase (*GPX*) of rabbits. Different superscript letters (a–c) on bars are meaningfully different at (*p* < 0.05) (*n* = 6).

The expressions of *SOD1* and *GPX* in the liver of rabbits fed dietary egg lysozyme and ZnB additives were markedly upregulated (*p* < 0.05) compared with the control (Figure 1). Dietary egg lysozyme resulted in higher expression of *SOD1* and *GPX* genes than rabbits treated with ZnB (*p* < 0.05).

## 4. Discussion

It becomes necessary to replace antibiotics with natural functional substances in the animal production industry to avoid the negative impacts on animals and human health [32,33,34]. Rabbit farming is a widespread activity providing humanity with delicious animal protein sources, but is threatened with infectious diseases involving high mortality rates and severe economic loss [35]. Traditionally, antibiotics are used to relieve the impacts of infection on rabbits’ health and well-being [36]. Alternatively, several functional additives such as probiotics, prebiotics, herbal extracts, and egg lysozyme have been included in rabbit feed to reduce antibiotics usage [37,38,39]. The inclusion of egg lysozyme successfully enhanced the growth rate, antibacterial capacity, and health status in rabbits in previous studies. El-Deep et al. [3] concluded that using egg lysozyme at 100–200 mg/kg is efficient for enhancing the performances of growing rabbits. Based on that, this study compared the positive effect of dietary lysozyme with zinc-bacitracin antibiotic (ZnB) in the feeds of rabbits.

The results show increased growth performance in rabbits treated with dietary lysozyme or ZnB, but rabbits fed lysozyme had superior enhanced growth performance. The results agree with El-Deep et al. [3], who stated that dietary egg lysozyme resulted in enhanced growth performance of growing rabbits. Furthermore, May et al. [16] and Oliver and Wells [18] reported that dietary egg lysozyme resulted in improved growth in weaning pigs. Additionally, Thema et al. [5] concluded that using ZnB in rabbits’ feed resulted in the enhanced growth performance of rabbits. The authors correlated enhanced growth performance with improved intestinal antibacterial capacity, leading to activated intestinal immunity with high absorption capacity. Both lysozyme and ZnB additives had antibacterial ability with increased protection against secretions of harmful microorganisms in the gut of animals [14,25]. Indeed, the high abundance of beneficial bacteria populations resulted in improved digestion of feeds and accommodated absorption of nutrients through the intestinal barriers to the bloodstream [40]. Interestingly, the FCR of rabbits treated with dietary lysozyme and ZnB was markedly reduced. The results agree with El-Deep et al. [3], who reported reduced FCR in rabbits treated with lysozyme. Low FCR value means that rabbits efficiently utilized feed in relation to the growth performance [41]. The superiority of dietary egg lysozyme compared to ZnB in enhancing the growth performance is probably associated with the influence of lysozyme on improving the FCR. It has been illustrated that lysozyme is responsible for protein synthesis in the blood through the binding of threonine, methionine, and hydroxyproline, resulting in high feed utilization and growth performance [42]. On the other hand, ZnB contains several functional polypeptides such as bacitracin A, B, and C with high growth-promoting activity [42].

The enhancement of feed utilization and growth rate is also associated with improved cecal microbial diversity, especially in beneficial populations [43,44]. The results show a high abundance of total bacterial count and *Lactobacillus* count along with decreased *Clostridium* spp. and *Escherichia coli* populations in rabbits treated with dietary egg lysozyme or ZnB. Similarly, rabbits treated with lysozyme showed high total bacterial and *Lactobacillus* counts but lowered *Clostridium* spp. and *E. coli* counts [3]. Brundige et al. [42] and Gong [45] also indicated that pigs treated with lysozyme had a lowered *E. coli* count. In the same line, rabbits treated with ZnB showed high antibacterial activity against *Clostridium* perfringens [25]. Egg lysozyme and ZnB have antibacterial activity against harmful bacteria, probably due to the role of these additives in hydrolyzing the peptidoglycan layer in bacterial cell walls [28]. Ibrahim et al. [28] discussed the antibacterial effect of egg lysozyme and proposed that peptides within the lysozyme sequence can attack the pathogenic bacteria in the gastrointestinal tract through two different modes of action—“dissipation of bacterial respiration and loss of membrane integrity”. Increased count of beneficial bacteria (*Lactobacillus*) and decreased *Clostridium* spp. and *E. coli* counts in this study explain the enhanced feed utilization and thereby growth performance in rabbits treated with lysozyme and ZnB. Herein, the enhancement of feed utilization can be related to the lack of pathogenic bacteria, resulting in an increased number of beneficial bacteria known for their ability to produce digestive enzymes to facilitate the digestion of nutrients in rabbits’ intestines [46]. In addition, the low number of harmful bacteria may lead to enhanced epithelial cell activity and the crossing of digested nutrients to the bloodstream. Generally, the lack of pathogenic bacteria and increased beneficial ones could activate local intestinal digestion and immunity, thereby activating the entire body’s immunity [41]. However, further studies are required to explain the mode of action of lysozyme in improving the antimicrobial capacity in rabbits’ intestines.

The evaluation of blood biochemical traits helps in testing the influence of dietary additives on the health status of animals [47,48]. Dietary egg lysozyme and ZnB are known for their growth-promoting and immunomodulation effects in rabbits [3]. Thus, neither egg lysozyme nor ZnB has harmful impacts on the blood metabolites (proteins and lipids), liver function (ALT and AST), and renal function (urea and creatinine). These results were observed under the current trial conditions, indicating the beneficial roles of egg lysozyme and ZnB on rabbits’ well-being. Similar results were seen by El-Deep et al. [3], who indicated that rabbits treated with egg lysozyme had enhanced blood proteins and regulated liver and kidney-related metabolites. Furthermore, Thema et al. [5] indicated that rabbits treated with ZnB had increased blood total proteins. The authors interpreted the increased blood proteins as a direct result of enhanced feed utilization and metabolic function in the entire body of rabbits. The regulation of kidney and liver function by dietary egg lysozyme and ZnB indicates the potential role of both additives in maintaining the health condition and well-being of growing rabbits. In addition, no abnormal features were observed in the blood lipid metabolites (cholesterol and triglycerides) in rabbits treated with egg lysozyme and ZnB, indicating the safe use of these additives.

Stressful farming conditions induce oxidative stress in rabbits due to the induction of reactive oxygen metabolites (ROS), leading to high lipid peroxidation [49,50]. In this sense, cellular defensive tools such as immune cells and antioxidative enzymes counteract the impacts of oxidative stress and degenerate ROS [51,52]. The hepatic tissue can metabolize harmful compounds involved in the formation of ROS. Hepatic antioxidative capacity is responsible for the degeneration of excessive ROS, resulting in balanced oxidative/antioxidative action [53]. In case of unhealthy liver status induced by stress, malnutrition, and toxicity, the liver antioxidative capacity is disrupted, leading to oxidative stress [54]. *SOD1* is the most abundant isoenzyme in the cellular cytoplasm, which reduces the superoxide radical anions, while *GPX* is another antioxidant enzyme involved in modifying harmful peroxides to non-harmful hydroxyl compounds to protect cellular structure and function [55]. The antioxidative enzymes include *SOD1* and *GPX*, which help in protecting the entire body and immune cells from oxidation [56,57]. The results show upregulation of antioxidant-related genes (*SOD1* and *GPX*) in the liver of rabbits treated with egg lysozyme and ZnB. The upregulation of *SOD1* and *GPX* genes is probably attributed to the role of egg lysozyme and ZnB additives in improving intestinal immunity [58]. It has been illustrated that egg lysozyme can improve antibody production in the intestinal mucosa of pigs, referring to the enhanced immunity and antioxidative capacity [18]. 

## 5. Conclusions

The results show marked enhancement of the growth performance, antibacterial capacity, blood health, and antioxidative status in rabbits treated with egg lysozyme comparing with those treated with ZnB. Thus, using egg lysozyme is recommended to replace the usage of ZnB in rabbit production.

## Figures and Tables

**Table 1:** Composition and chemical analysis (dry matter basis) of the basal diet.

Ingredients	%	Analysis	%
Berseem hay	30	Dry matter	89.7
Barley	23	Crude protein	17.2
Soybean meal	17.5	Crude fiber	13.1
Wheat bran	15	Ether extract	3.4
Yellow corn	10	Nitrogen free extract	56.
Molasses	3	Ash	10.3
Di. Ca. phosphate	0.8	Digestible energy (Kcal/kg)	2519
Sodium chloride	0.3		
Vitamin and mineral premix *	0.3		
DL-Methionine	0.1		

* The mixture provides per kilogram contains vitamin A 6000 IU, vitamin D_3_ 450 IU, vitamin E 40 mg, vitamin K_3_ 1 mg, vitamin B_1_ 1 mg, vitamin B_2_ 3 mg, vitamin B_3_ 180 mg, vitamin B_6_ 39 mg, vitamin B_12_ 2.5 mg, pantothenic acid 10 mg, biotin 10 mg, folic acid 2.5 mg, choline chloride 1200, manganese 15 mg, zinc 35 mg, iron 38 mg, copper 5 mg, selenium 0.1 mg and iodine 0.2 mg.

**Table 2:** Primer design used in the study.

Gene	Sequence Primer (5’–3’)	Accession Number
*GAPDH*	F: GGAGAAAGCTGCTAA	L23961
	R: ACGACCTGGTCCTCGGTGTA	
*SOD1*	F: CGCCGCTCGGAAGTCAT	XM_017347219
	R: TTATGCGTCCCTTGACCACC	
*GPX1*	F: TTCGAGCCCAACTTCATGCT	NM_001085444
	R: TCGAAGCTCCAGGAAACGTC	

Glyceraldehyde 3-phosphate dehydrogenase (*GAPDH*), superoxide dismutase (*SOD1*) and glutathione peroxidase (*GPX*).

**Table 3:** Effect of chicken egg lysozyme (LZM) and zinc bacitracin antibiotic (ZnB) on the growth performance of rabbits.

	Control	ZnB	LZM
Initial BW (g)	612 ± 0.43 ^a^	611.25 ± 0.26 ^a^	611 ± 0.45 ^a^
Final BW (g)	2256 ± 0.33 ^c^	2273 ± 0.91 ^b^	2343 ± 0.71 ^a^
BWG (g)	1644 ± 0.80 ^c^	1662 ± 0.24 ^b^	1732 ± 0.30 ^a^
FI (g, as feed)	5490.96 ± 32.55 ^a^	4853.04 ± 41.69 ^b^	4676.4 ± 65.22 ^c^
Total FCR	3.34 ± 0.02 ^a^	2.92 ± 0.11 ^b^	2.70 ± 0.05 ^c^

Different superscript letters (^a–c^) within the same row are meaningfully different at (*p* < 0.05) (*n* = 30). BW = bodyweight (g), BWG= body weight gain (g), FI= feed intake (g, as feed), FCR= feed conversion ratio.

**Table 4:** Effect of chicken egg lysozyme (LZM) and zinc bacitracin antibiotic (ZnB) on intestinal microbial load of rabbits.

	Control	ZnB	LZM
*Clostridium* spp.	6.11 ± 0.05 ^a^	4.58 ± 0.01 ^b^	4.14 ± 0.05 ^b^
*Escherichia coli* (×10^4^)	1.61 ± 0.02 ^a^	1.02 ± 0.02 ^b^	1.11 ± 0.11 ^b^
*Lactobacillus* (×10^5^)	5.08 ± 0.04 ^c^	9.84 ± 0.02 ^b^	10.76 ± 0.02 ^a^
Total bacterial count (×10^6^)	16.5 ± 0.01 ^c^	19.84 ± 0.02 ^b^	24.7 ± 0.02 ^a^

Different superscript letters (^a–c^) within the same row are meaningfully different at (*p* < 0.05) (*n* = 6). Counts were presented in CFU/g cecal digesta.

**Table 5:** Effect of chicken egg lysozyme (LZM) and zinc bacitracin antibiotic (ZnB) on blood biochemical variables of rabbits.

	Control	ZnB	LZM
Total protein (g/dL)	6.30 ± 0.25 ^c^	7.03 ± 0.03 ^b^	7.46 ± 0.32 ^a^
Albumin (g/dL)	4.05 ± 0.24 ^a^	3.71 ± 0.11 ^a^	3.7 ± 0.12 ^a^
Globulin (g/dL)	2.25 ± 0.06 ^c^	3.33 ± 0.14 ^b^	3.76 ± 0.12 ^a^
Albumin/globulin ratio	1.80 ± 0.15 ^a^	0.89 ± 0.17 ^a^	0.98 ± 0.10 ^a^
Creatinine (mg/dL)	1.18 ± 0.20 ^a^	1.10 ± 0.04 ^a^	0.87 ± 0.12 ^b^
Urea (mg/dL)	4.40 ± 3.29 ^a^	3.67 ± 1.45 ^b^	3.53 ± 0.67 ^b^
Aspartate aminotransferase (AST) (IU/L)	55.0 ± 1.53 ^a^	53.67 ± 1.20 ^b^	51.3 ± 0.88 ^c^
Alanine aminotransferase (ALT) (IU/L)	47.0 ± 1.53 ^a^	35.67 ± 1.20 ^b^	34.6 ± 0.88 ^b^
Cholesterol (mg/dL)	28.3 ± 1.77 ^a^	26.00 ± 1.53 ^a^	28.7 ± 0.58 ^a^
Triglyceride (mg/dL)	74.7 ± 0.88 ^a^	83.67 ± 0.88 ^a^	73.3 ± 1.73 ^a^

Different superscript letters (^a–c^) within the same row are meaningfully different at (*p* < 0.05) (*n* = 9).

## Data Availability

The datasets generated during and/or analysed during the current study are available from the corresponding author on reasonable request.
